# Supplementary material for: Association between antenatal care utilization pattern and timely initiation of postnatal care checkup: Analysis of 2016 Ethiopian Demographic and Health Survey
Source: PLoS One. 2021 Oct 12;16(10):e0258468. doi: 10.1371/journal.pone.0258468 (PMC8509870; doi:10.1371/journal.pone.0258468)
Supplement: Attachment — Submitted filename: Response to Reviewers.docx [file pone.0258468.s001.docx]

**Response to the Reviewers**

Dear editor, thank you for considering our manuscript for publication; we have revised the contents based on the reviewer’s comments as below.

Ref: Submission ID : PONE-D-20-37280
"Association between antenatal care utilization pattern and timely initiation of postnatal care checkup: Analysis of 2016 Ethiopian Demographic and Health Survey
PLOS ONE."

Additional Editor Comments:

To relook into the linkage between the use of antenatal care and postnatal care. And among those with postnatal care, to differentiate between home and institutional deliveries. And the connection with these two settings for childbirth.

*Response: We have adjusted for place of delivery in the analysis.*

[Note: HTML markup is below. Please do not edit.]

Reviewers' comments:

Reviewer's Responses to Questions

**Comments to the Author**

1. Is the manuscript technically sound, and do the data support the conclusions?

The manuscript must describe a technically sound piece of scientific research with data that supports the conclusions. Experiments must have been conducted rigorously, with appropriate controls, replication, and sample sizes. The conclusions must be drawn appropriately based on the data presented.

Reviewer #1: Partly

2. Has the statistical analysis been performed appropriately and rigorously?

Reviewer #1: Yes

3. Have the authors made all data underlying the findings in their manuscript fully available?

The [PLOS Data policy](http://www.plosone.org/static/policies.action#sharing) requires authors to make all data underlying the findings described in their manuscript fully available without restriction, with rare exception (please refer to the Data Availability Statement in the manuscript PDF file). The data should be provided as part of the manuscript or its supporting information, or deposited to a public repository. For example, in addition to summary statistics, the data points behind means, medians and variance measures should be available. If there are restrictions on publicly sharing data—e.g. participant privacy or use of data from a third party—those must be specified.

Reviewer #1: Yes

4. Is the manuscript presented in an intelligible fashion and written in standard English?

PLOS ONE does not copyedit accepted manuscripts, so the language in submitted articles must be clear, correct, and unambiguous. Any typographical or grammatical errors should be corrected at revision, so please note any specific errors here.

Reviewer #1: Yes

5. Review Comments to the Author

Please use the space provided to explain your answers to the questions above. You may also include additional comments for the author, including concerns about dual publication, research ethics, or publication ethics. (Please upload your review as an attachment if it exceeds 20,000 characters)

Reviewer #1: General comments:
The authors have highlighted a critical issue in Ethiopia: low prevalence of timely use of postnatal care. They hypothesize an association between antenatal care and postnatal care and use a nationally representative dataset to test that association. However, the paper is missing a critical analysis and discussion of the expected pathway/linkages between use of antenatal care and postnatal care; this re-analysis and re-write would be necessary in order to make useful recommendations for future study or intervention.

Response: Thank you.

Major comments (should be addressed before publication)

1. The manuscript would benefit from a detailed description of what the authors think the theory of effect is here. How would ANC affect PNC? Are they both driven by a confounder that affects access? Is the experience of ANC (e.g. quality of care) driving the decision to go for PNC? Are current covariates included because they are confounders or just trying to soak up variation? A description and a DAG would be helpful for understanding the importance of this analysis, what it might mean, and for the analytic choices made.
a. Note, assessing counseling as a predictor of PNC could provide more strength to the hypothesis shared in the discussion section :“Because ANC is a time when mothers are educated about childbirth and childcare practices, those who follow the ANC guidelines might be better informed about PNC guidelines.”
b. Also in the discussion, the following assertion can be tested using DHS data and therefore should be tested if you want to discuss it in the discussion: “Increased numbers of antenatal visit might give opportunities to women to learn about birth preparedness, potential complications during pregnancy and labor”

***Response:*** *We appreciate this feedback.*

*a. As indicated in the introduction section we tested the hypothesis that women who had timely and at least four ANC visits would be more likely to have a timely PNC checkup. Covariates were selected based on existing literature on factors influencing timely PNC checkup. We have now indicated this in the methods section (Page 6, Line 151-152). As the reviewer indicated precisely, we were testing if antenatal counselling is associated with timely PNC checkup.*

*b. This was one possible explanation for the observed relationship between number of antenatal care visit and increased likelihood of PNC (Page 13, Line 227-230).*

2. Given the very large difference between those who delivered in an institution versus home, I would recommend stratifying those populations. Who got a postnatal visit among those who delivered in an institution, and who among those who delivered at home? This ties again into the theory of change – does ANC lead more women to deliver in an institution, which then leads them to have a timely PNC visit, or does ANC lead to increased PNC regardless of institutional delivery? Note that this phrasing is causal, because I’m thinking about theory, but this analysis will only look at associations not causation.

***Responses:*** *Thank you for the feedback.* *As you stated clearly this study examined the association between ANC number and timing with timely receipt of PNC.* *By controlling place of delivery in the multivariable regression model we are taking these differences into account. We were not interested in effect measure modification by place of delivery, which of course can be a separate manuscript by itself.*

3. Again, depending on your theory of change, it is likely that one pathway through which ANC could affect PNC use is through quality of care (the authors allude to this in the last paragraph of the introduction). The analysis would be strengthened if this hypothesis were tested as well. The authors could create a quality of care index based on the services reported as received during ANC (while patient report is not the gold standard for these process of care measures, it might provide some indication of quality of care).

***Response:*** *Thank you for this feedback. The focus of our research was the timing and number of ANC visits as recommended by the World Health Organization (WHO), i.e., least four ANC visits during pregnancy and first ANC during the first trimester. Assessing the components on ANC was out of scope for this manuscript.*

4. Discussion: please discuss how 36.9% of women gave birth in the health facility, but only 16.5% had a postnatal check-up within two days.

Are women being discharged without a postnatal check-up?

***Response:*** *Ideally, postnatal care is best delivered in a health facility. However, in Ethiopian context due to many socio-economic and cultural reasons, such as the lack of MNH service, lack of trained professional, lack of bed and essential medicines women are obligated to be discharged early without a postnatal checkup to get space for the complicated ones. Therefore, it is likely that some women are discharged without postnatal check-up.*

5. How long after delivery does the checkup have to occur to be considered “postnatal”?

***Response:*** *Postnatal care (PNC) is the care given to the mother and her newborn baby immediately after the birth and for the first six weeks of life. In the current study we used WHO’s 2018 Global reference list of 100 core health indicators (‎plus health-related SDGs)‎ to define the timely PNC as proportion of women who have postpartum contact with a health provider within 2 days of delivery (Page 5, Line 142-143).*

6. This second question ties into the confusion above of discussing hemorrhage and birth asphyxia in relation to postnatal care. It would be helpful to see the question from the DHS that this analysis is based on (does it mean that ~half of the people who delivered in the facility answered “no” to the question “Did anyone check on your health while you were still in the facility?” The authors discuss that women are supposed to stay for 24 hours, so this issue of women who deliver in a health facility not receiving a PNC visit seems to be one of quality of care, not of the woman’s choice or access.

***Response:*** *Even though the recommendations given by WHO women are supposed to stay at health facility for 24 hours, practically women discharged on average for six hours. This is mainly due to lack of space. However, women are told to come back for postnatal checkup. This may be due to sub-standard quality of service practiced by health professionals as you mentioned.*

5. The paper focuses entirely on the first postnatal visit – is it possible to also look at subsequent visits?

***Response:*** *This is possible. However, the focus of this paper was timely initiation of postnatal care; defined with the current WHO indicators as within two days after delivery. We also wanted to focus on this period because around 45-50% of mothers and newborns die in the first 24 hours after birth.* *So, if PNC is initiated early, many unforeseen complications may be identified and intervene early.*

6. Together with the careful theory of change discussion requested above, I think the paper would benefit from a careful read to make sure there is no causal language implied. Without understanding the pathways through which ANC and PNC are associated, it’s hard to know if increasing ANC would meaningfully impact PNC (the authors do include this in their limitations section, which is great).

***Response:*** *Thank you. As we stated in the introduction, we were interested in assessing the hypothesis that receipt of the recommended number of ANC may influence timely PNC.*

Minor comments (to improve clarity)

1. Introduction: the first and second sentence almost seem at odds with one another. Hemorrhage and birth asphyxia within the first day would be most affected by delivery at a health facility. Maybe adding other causes of morbidity and mortality that would also be affected by the subsequent postnatal visits would be helpful.

***Response:*** *We have added some other complications /morbidities which occur following delivery.*

2. Figure 1: It’s helpful to break out the women who were not eligible (didn’t deliver in past two years) from those who had.

***Response:*** *Corrected as recommended (figure 1).*

3. Table 1: Is education the highest level completed? Please clarify in the table

***Response:*** *Yes, it is the highest educational level. We have changed the label in the tables to reflect this.*

4. Sample characteristics: The sentence “Nearly half (42.2%) of the women were multiparous (had two or more pregnancy).” is misleading. I suggest reporting the number of primiparous women (preferable), or report the full number of multiparous, which according to the table would be 42.2%+37.4%

***Response:*** *We have corrected as recommended.*

5. Typo in the aOR for secondary education, missing the decimal place (164)

***Response:*** *Thank you. We have corrected this to read as corrected as 1.64.*

6. Discussion: this sentence seems to confuse multiparous with grand multiparous, which appears to be the comparison group in table 1 “Primiparous women were 66 % less likely to utilize postnatal checkup timely as compared with multiparous women.”

***Response:*** *Thank you. We have corrected this as recommended.*
