# Supplementary material for: Association between antenatal care utilization pattern and timely initiation of postnatal care checkup: Analysis of 2016 Ethiopian Demographic and Health Survey
Source: PLoS One. 2021 Oct 12;16(10):e0258468. doi: 10.1371/journal.pone.0258468 (PMC8509870; doi:10.1371/journal.pone.0258468)
Supplement: Attachment — Submitted filename: Response to Reviewers.docx [file pone.0258468.s002.docx]

**Response to the Reviewers**

Dear editor, thank you for considering our manuscript for publication; we have revised the contents based on the reviewer’s comments as below.

Ref: Submission ID : PONE-D-20-37280
"Association between antenatal care utilization pattern and timely initiation of postnatal care checkup: Analysis of 2016 Ethiopian Demographic and Health Survey
PLOS ONE."

Reviewers' comments:

Reviewer's Responses to Questions

Comments to the Author

1. If the authors have adequately addressed your comments raised in a previous round of review and you feel that this manuscript is now acceptable for publication, you may indicate that here to bypass the “Comments to the Author” section, enter your conflict of interest statement in the “Confidential to Editor” section, and submit your "Accept" recommendation.

Reviewer #2: (No Response)

Reviewer #3: All comments have been addressed

2. Is the manuscript technically sound, and do the data support the conclusions?

The manuscript must describe a technically sound piece of scientific research with data that supports the conclusions. Experiments must have been conducted rigorously, with appropriate controls, replication, and sample sizes. The conclusions must be drawn appropriately based on the data presented.

Reviewer #2: Yes

Reviewer #3: Yes

3. Has the statistical analysis been performed appropriately and rigorously?

Reviewer #2: Yes

Reviewer #3: Yes

4. Have the authors made all data underlying the findings in their manuscript fully available?

The PLOS Data policy requires authors to make all data underlying the findings described in their manuscript fully available without restriction, with rare exception (please refer to the Data Availability Statement in the manuscript PDF file). The data should be provided as part of the manuscript or its supporting information, or deposited to a public repository. For example, in addition to summary statistics, the data points behind means, medians and variance measures should be available. If there are restrictions on publicly sharing data—e.g. participant privacy or use of data from a third party—those must be specified.

Reviewer #2: Yes

Reviewer #3: Yes

5. Is the manuscript presented in an intelligible fashion and written in standard English?

PLOS ONE does not copyedit accepted manuscripts, so the language in submitted articles must be clear, correct, and unambiguous. Any typographical or grammatical errors should be corrected at revision, so please note any specific errors here.

Reviewer #2: Yes

Reviewer #3: Yes

6. Review Comments to the Author

Please use the space provided to explain your answers to the questions above. You may also include additional comments for the author, including concerns about dual publication, research ethics, or publication ethics. (Please upload your review as an attachment if it exceeds 20,000 characters)

Reviewer #2: The authors have effectively addressed the majority of the reviewer comments. However, a few important concerns remain unaddressed. These include the following:

1. An important comment by the reviewer "A description and a DAG would be

helpful for understanding the importance of this analysis, what it might mean, and for the analytic choices made" was not addressed.

***Response:*** *Thank you for the feedback. We understand that DAGs can be used map all a priori assumptions surrounding a causal question of interest and identify potential confounders. In the current study we used one of the conventional approaches i.e., selecting potential socio-demographic and obstetric confounders based on previous research which have been cited in the study variables section.*

2. On the sample characteristics, the comment "Sample characteristics: The sentence “Nearly half (42.2%) of the women were multiparous (had two or more pregnancy) ” is misleading" was not addressed as claimed by the authors. Here, I believe the reviewer wanted authors to directly state 42.2% as this figure cannot be considered as "nearly half".

***Response:*** *"nearly half (42.2%) “has been changed to “About 42.2%”*

3. The "Highest educational level" in Table 1 should also be replicated in Tables 2 and 3 as well as under the "Study variable".

***Response:*** *Thank you for the feedback. We have made these corrections in tables 2 and 3.*

4. This is very important. Some of the journal format guidelines were not adhered to by the authors. For example,

the in-text citations are expected to be in square brackets but not round. Ultimately, reference no. 4 "postpartum-complications[1]" was poorly cited and should be checked and fully provided. This also applies to reference numbers 21 (https://doi.org/10.11564/28-3-638) and 22 in the reference list.

Finally, the journal requires authors to provide the "Declarations" (Authors’ contributions, funding, competing interests, Availability of data and materials among others) in the editorial manager system but not in the manuscript as provided by the authors. Authors have to address this to avoid possible delay in the publication of their paper.

***Response:*** *Thank you for your detail review and feedback. We have checked and recited (reff #4, 15, 21&22).*

*We have now used the Vancouver referencing style using square brackets for the in-text citations as indicated in the authors guideline for the journal.* *.*

*We have also removed the "Declarations" (Authors’ contributions, funding, competing interests, Availability of data and materials among others) from the manuscript as recommended.*

Reviewer #3: Authors have carefully addressed almost all the issues raised by reviewers in the first review process. However, the additional comments below should be addressed to enhance the quality of the paper.

General comments:

There are some few grammatical errors in the manuscript including missing commas, missing function words and dots. E.g. (lines; 41, 75, 82-83, 136, 175-175, 217 etc.). Authors should carefully read through the work and correct all grammatical errors in the papers.

***Response:*** *Thank you for the feedback. We revised and corrected per your suggestions (lines; 41, 75, 82-83, 136, 175-175, 217).*

The sentence on line 41-42 should be changed to “Chi-square test and multivariable logistic regression analyses were used to...”

***Response:*** *Thank you. This has been corrected as recommended. .*

The sentence on line 164 should also be changed to “Multiple logistic regression analyses was performed…”

***Response:*** *Thank you for the feedback. Corrected (line 164).*

Authors should avoid the use of complex sentences which are difficult to understand e.g. (lines 88-89)

***Response:*** *Rephrased (lines 88-89)*

Authors should be clear on the marital status of participants. What does the “Yes” and “No” categories of the marital statuses of participants in the results represent? E.g. Married or Single

The “None” as a category of educational status in the result section should be changed to “No formal education”

The sentence on line 202- 203 should be changed to …women who had secondary and above education (aOR: 1.64; 95%CI: 1.03-2.60) compared to those who had no formal education…”

***Reponses:*** *Thank you for the feedback. All the above issues have been addressed as recommended (Tables 1, 2, 3).*

Authors should briefly discuss how similar or different their study results are in relation to other studies. Thus, what are the evidence in literature of which results in this study is consistent with? (Lines 217-218, lines 231-232, line 239, lines 245-246, line 254

*Response: We appreciate this feedback. Discussions have been added regarding the consistency of the findings with existing literature.*
