# Supplementary material for: Association between antenatal care utilization pattern and timely initiation of postnatal care checkup: Analysis of 2016 Ethiopian Demographic and Health Survey
Source: PLoS One. 2021 Oct 12;16(10):e0258468. doi: 10.1371/journal.pone.0258468 (PMC8509870; doi:10.1371/journal.pone.0258468)
Supplement: Attachment — Submitted filename: Response to Reviewers.docx [file pone.0258468.s003.docx]

**Response to the Reviewers**

Dear editor, thank you for considering our manuscript for publication; we have revised the contents based on the reviewer’s comments as below.

Ref: Submission ID : PONE-D-20-37280
"Association between antenatal care utilization pattern and timely initiation of postnatal care checkup: Analysis of 2016 Ethiopian Demographic and Health Survey
PLOS ONE."

Journal Requirements:

Please review your reference list to ensure that it is complete and correct. If you have cited papers that have been retracted, please include the rationale for doing so in the manuscript text, or remove these references and replace them with relevant current references. Any changes to the reference list should be mentioned in the rebuttal letter that accompanies your revised manuscript. If you need to cite a retracted article, indicate the article’s retracted status in the References list and also include a citation and full reference for the retraction notice.

*Response: We have reviewed our reference list. No retracted papers were identified.*

Additional Editor Comments (if provided):

Language checking and thorough editing is recommended.

*Response: We have edited the manuscript for language.*

[Note: HTML markup is below. Please do not edit.]

Reviewers' comments:
